# Effects of continuous moderate exercise with partial blood flow restriction during hemodialysis: A protocol for a randomized clinical trial

**DOI:** 10.1016/j.mex.2019.01.005

**Published:** 2019-01-23

**Authors:** Rodrigo Kohn Cardoso, Aline Machado Araujo, Rafael Bueno Orcy, Maristela Bohlke, Jean Pierre Oses, Fabrício Boscolo Del Vecchio, Franklin Correa Barcellos, Maria Cristina Gonzalez, Airton José Rombaldi

**Affiliations:** a Post-Graduate Program of Physical Education, Federal University of Pelotas, Pelotas, RS, Brazil; b Department of Physiology and Pharmacology, Federal University of Pelotas, Pelotas, RS, Brazil; c Dialysis and Renal Transplantation Unit, São Francisco de Paula University Hospital, Catholic University of Pelotas, Pelotas, RS, Brazil; d Post-Graduate Program in Health and Behavior, Catholic University of Pelotas, Pelotas, RS, Brazil

**Keywords:** Chronic kidney failure, Hemodialysis, Rehabilitation, Training, Inflammation, Oxidative stress, Quality of life

## Abstract

Chronic kidney disease (CKD) is associated with physical weakness and increased oxidative stress and inflammation levels. Rehabilitation programs are associated with an improvement in the functional capacity, inflammatory and oxidative stress profile. Exercise associated with blood flow restriction (BFR) has been demonstrating positive effects in training programs, but there is lack information about exercise with BFR in CKD. Therefore, the aim of the present study is to describe a protocol using continuous moderate exercise with blood flow restriction (BFR) applied during hemodialysis (HD) to measures health indicators and immune system and oxidative stress parameters in CKD patients. Methods: A RTC will be conducted with 42 patients in HD. Baseline measures will be compared with final measures (anthropometric, cardiorespiratory, biochemical, muscle fitness, nutritional and behavioral questions). Participants will be randomly allocated to: 1) Continuous moderate exercise group with BFR; 2) Continuous moderate exercise group without BFR; 3) Control group without exercise. The intervention will be 12 weeks long during HD session. Patients will perform 20 min of continuous moderate exercise on a stationary bicycle three times a week. The present study is expected to generate significant information about the role of exercise with BFR in patients with CKD during HD.

## Graphical abstract

**Figure:**
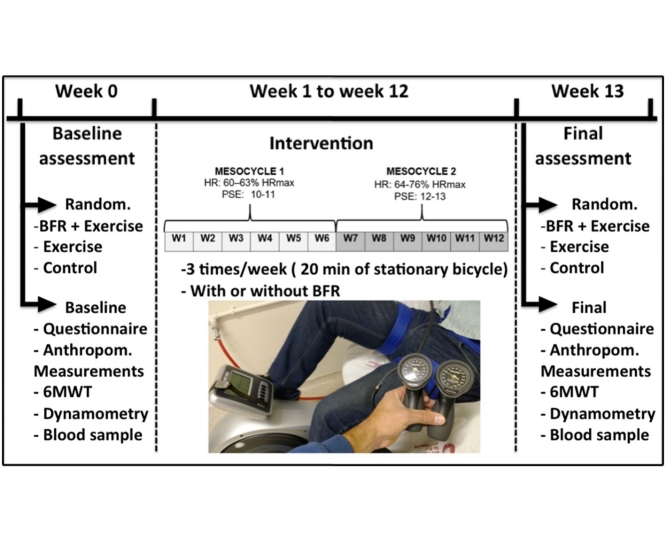

**Specifications Table**

**Table:** 

**Subject Area**	• *Medicine and Dentistry*
**Reagents/tools:**	- *Ergometer cycle Oneal b2901d2* - *Cuff BFR Sistem (Hokanson)*
*Experimental design:	Experimental randomized controlled trial (RCT), open-label, phase III-type study with 42 patients in HD. Baseline measures will be compared with final measures (anthropometric, cardiorespiratory, biochemical, muscle fitness, nutritional and behavioral questions). Participants will be randomly allocated to: 1) Continuous moderate exercise group with BFR; 2) Continuous moderate exercise group without BFR; 3) Control group without exercise. The intervention will be 12 weeks long during HD session. Patients will perform 20 minutes of continuous moderate exercise on a stationary bicycle three times a week during HD session The exercise will be performed in the first two hours of HD to avoid hypotension effects that occur usually in the second half of the 4-hour session.
**Trial registration:**	The study protocol was registered in the Brazilian Registry of Clinical Trials, under protocol RBR-8T2P2M.
**Ethics:**	The study protocol was submitted to the Research Ethics Committee of the Catholic University of Pelotas and approved under No. 2,036,385.
*Value of the Protocol:	- This protocol is important to verify the effects of moderate intensity aerobic exercise associated with BFR. - There is a lack of knowledge about the effects of BFR in chronic diseases, and this work will help elucidate important points. - This rehabilitation protocol have a especific training program and will yield important results regarding the effects on the inflammatory profile, markers of oxidative stress and funcional capacity in chronic kidney patients.

## Description of protocol

Chronic kidney disease (CKD) is associated with physical weakness and increased oxidative stress and inflammation levels [1,2]. Rehabilitation programs are associated with an improvement in the functional capacity, inflammatory and oxidative stress profile.2] Exercise associated with blood flow restriction (BFR) has been demonstrating positive effects in training programs, but there is lack information about exercise with BFR in CKD [[3], [4], [5]]. Therefore, the aim of the present study is to describe of a protocol using continuous moderate exercise with blood flow restriction (BFR) applied during hemodialysis (HD) to measures health indicators and immune system and oxidative stress parameters in CKD patients.

### Design

An experimental randomized controlled trial (RCT), open-label, phase III-type study will be conducted, the SPIRIT (Standard Protocols Items: Recommendations for Interventional Trials) recommendations were followed, to evaluate the effectiveness of the BFR intervention. In addition, the data collection and the statistical analysis will be carried out by people unaware of the objectives of the study.

### Subjects

HD patients from the Nephrology unit of the Hospital São Francisco de Paula in Pelotas, Southern Brazil, users of the Brazilian Health System (SUS), aged 18 years and older from both sexes.

### Exclusion criteria

For the exclusion criteria, we list possible associated pathologies that limit or are contraindicated for exercise prescription.

a) Diagnosis of coronary artery disease, presence of active infection or cancer;
b) Presence of musculoskeletal limitations preventing exercise performance;
c) Cognitive alterations making it impossible to understand the instructions of the exercises;
d) Systolic blood pressure ≥ to 180 mmHg or diastolic blood pressure ≥105 mmHg at rest; resting heart rate ≥ to 120 bpm.

### Sample size calculation

Sample size was calculated to estimate the occurrence of outcomes in each of the groups separately and to evaluate the association between each outcome: muscle thickness [5], IL-6 [6], TNF-alfa [7] and, quality of life [8], and the exposure variable. According to the largest sample size calculation, 36 patients would be required (to maintain a minimum power of 80% and a 95% confidence level). To compensate for possible losses and refusals, 42 patients will be included [[5], [6], [7], [8]].

### Recruitment

Subjects will be recruited at the nephrology department, all chronic patients of the unit who is on hemodialysis treatment will be investigated in your medical records and basic information will be analyzed allowing the application of some exclusion criteria. Afterward, they will be individually interviewed and only who fill all criteria to be included, invited to participate in the study. Then, the goals of the study will be explained and the permission to perform the intervention will be solicited.

### Randomization

The randomization process will be performed using the generation of random numbers in the Stata software, version 13, and the researcher who will carry out the process will be blinded regarding to the study objectives. The randomization will be done in blocks, each block referring to the HD shift. In each block, the first patient drawn will go to the BFRG, the second to the EG, the third to the CG, and so on. In total, 14 patients will be drawn in each block to ensure balance between groups.

### Procedures

**Fig. 1:**
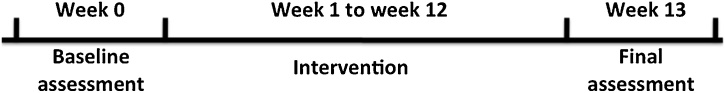
Intervention schedule.

Immediately after recruitment, all enrolled patients who agree to participate in the study will sign the informed consent form. The subjects will then proceed to the baseline data collection, intervention protocol and final assessments, respectively (Fig. 1).

The baseline measurements will be distributed at week 1 as follows. At the 1st HD session of the week the resting HR, resting blood pressure, ankle-arm index (AAI) measurements and questionnaire application will be performed. At the 2nd HD session of the week the percentage of fat, body mass and height measures, and blood collection will be obtained, after the HD session. At the 3rd HD session of the week the functional capacity (using the 6-minute walk test) and muscle strength (using dynamometry) will be measured. The final evaluations (post-intervention) will follow the same weekly distribution of the baseline measures.

Blood pressure will be measured with an accurate analog sphygmomanometer (accuracy of 1.0 mm/Hg) and stethoscope (BD, Brazil). Body mass and height will be measured using digital electronic anthropometric scale with a resolution of 0.1 kg and 0.1 cm, respectively (Filizola, Brazil). Body mass index (BMI) will be calculated based on body mass and height. The HR will be measured using a digital HR monitor (Polar, Finland).

To calculate ankle-arm index (AAI) the patient should rest supine for at least 10 min before testing, the systolic blood pressure measurement will be performed in the upper limb using an analog sphygmomanometer. The measure will be performed on the arm that is not with the dialysis fistula. The systolic blood pressure at the ankles will be measured using the vascular Doppler with transducer of 5–8 MHz with application of gel on the analyzed region and the same model of sphygmomanometer as previously reported [9]. The cuff will be placed about three inches above the malleolus. The following formula will be applied to calculate AAI: AAI = higher ankle pressure x higher arm pressure^−1^ [9].

The femoral quadriceps muscle thickness will be measured by MicroMaxx(Sonosite®) ultrasound, according to a protocol proposed by Paris et al. [10] Two reference points on the anterior surface of each quadriceps will be identified and marked with an indelible ink pen: the first one on the midpoint between the anterior superior iliac spine and the upper pole of the patella and the second on the border of the lower third and upper two thirds between the upper anterior iliac spine and the upper pole of the patella [9]. The muscle thickness will be quantified using the frozen image on the screen considering the distance between the upper margin of the femoral bone and the lower limit of the deep fascia of the *rectus femoris*, incorporating both *rectus femoris* and the *vastus medialis* skeletal muscles.

The percentage of fat will be estimated by body composition analysis using multifrequency bioelectrical impedance analysis (Quadscan4000, Bodystat®), according to recommendations and a protocol previously published by Kyle et al.: in supine body position, arms stretched along the trunk and legs apart. The electrodes will be placed respecting the distance of at least 5 cm on the previously cleaned skin using alcohol. The assessment will be performed 20 to 30 min after the middle-week HD session, being important to protocol the day and time of the measurements, as well as a period of a few minutes post-dialysis to balance the fluid concentrations of the body compartments [11]. The percentage of body fat will be estimated using the manufacturer’s equation.

Considering the measurement of body composition and quadriceps femoral muscle thickness, patients will be instructed to avoid physical exercise practice eight hours before and avoid consuming alcohol, chocolate, coffee, tea and energy drinks in the 12 hs prior to the exam. Body composition and muscle thickness will be measured between 20–30 min after the HD session, as recommended by Kyle et al. [11].

**Fig. 2:**
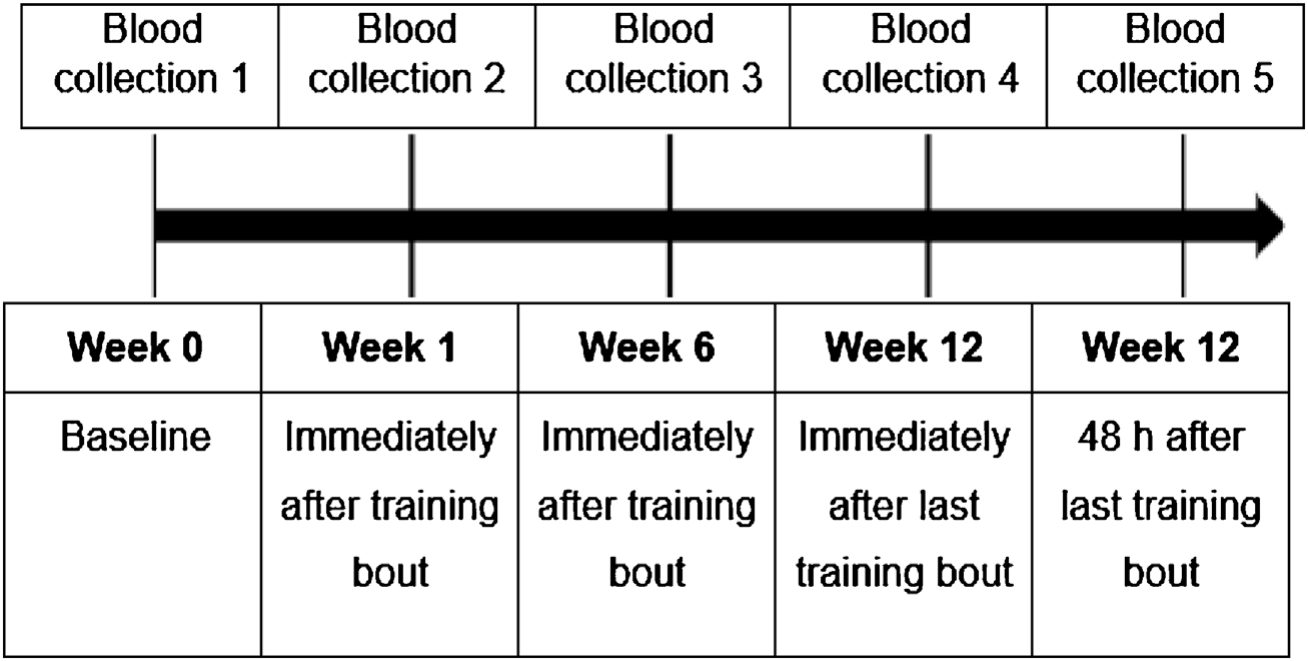
Schedule of blood collections.

A trained nurse will draw 10 mL of blood directly from intravenous fistula, 5 mL in the tube with clot activator and another 5 mL in the anticoagulant tube. The blood will be separated and stored. The erythrocytes fraction will be isolated and stored to oxidative stress analyses. Blood samples will be collected at baseline and repeated after the third training session (baseline blood lactate concentration after training) and after the end of the last training session of each training mesocycle, at the end of weeks six and twelve, respectively. The last blood collection will be performed 48 h after the last exercise session, to avoid acute effects of the exercise influencing inflammatory and oxidative stress markers (Fig. 2).

Serum levels of IL-6, IL-10, and CRP will be measured using immunoassay kit (DuoSet ELISA Development, R&D Systems, Inc., USA). These methods will follow the manufacturer's recommendations. The amount of IL-6, IL-10, and CRP will be determined by measuring the absorbance at 450 nm. All samples and standards will be measured in duplicate, and the coefficient of variation will be less than 5%. The IL-6, IL-10, and CRP levels will be expressed in pg/mL.

Oxidative stress markers (catalase, peroxide dismutase, and glutathione peroxidase enzymes) will be measured using a commercially available kit (RANSEL®; Randox Lab, Antrim, United Kingdom), using the method described by Misra and Fridovich [12].

The 6-minute walk test will be conducted on a 30-meter flat track, demarcated every three meters. Before starting the test, patients will remain seated for 10 min to stabilize vital signs. The patient will be advised to walk for 6 min, from one end of the lane to the other, at the highest possible speed. This test will be performed before the HD session, since patients cannot walk well after the HD session due to the loss of fluid with hypotensive effects [13].

The static strength testing of lower limbs will be performed with a Crown® brand dynamometer. Initially, patients will be instructed to stand on the base of the device, flexed knees forming an angle of approximately 120°, upright spine, arms along the body and extended elbows. After resetting the instrument, the subjects should perform the maximum knee extension force possible, avoiding any movement with the spine or arms and moving the body backwards. Two measurements will be performed with a 1 min interval between them. The mean of these two measures will be used as a result [14].

After baseline collection, participants will be allocated randomly: 1) BFRG; 2) EG; 3) CG. The intervention will last twelve weeks divided into three weekly sessions, always having at least a day interval between sessions. All physical exercise sessions will be monitored by physical therapists and physical education teachers to guarantee the perfect execution of the exercises and the correct use of the BFR accessory and heart monitor.

### Intervention description

The physical exercise groups – BFRG and EG – will be submitted to an exercise protocol composed by 20 min on the stationary bicycle. The exercise will be performed in the first two hours of HD to avoid hypotension effects that occur usually in the second half of the 4-hour session [1,3,15]. A cycle ergometer will be placed in front of the patient's chair and will be positioned at a distance allowing a relative knee angle (inner angle formed between the thigh and the leg) between 150° and 155° and using the following progression:

Mesocycle 1 – Weeks 1 to 6: participants will cycle at an intensity eliciting HR between 60 and 63% of HRmax or 10 to 11 in the perceived subjective exertion (PSE) scale, which ranges from 6 to 20 [16].

**Fig. 3:**
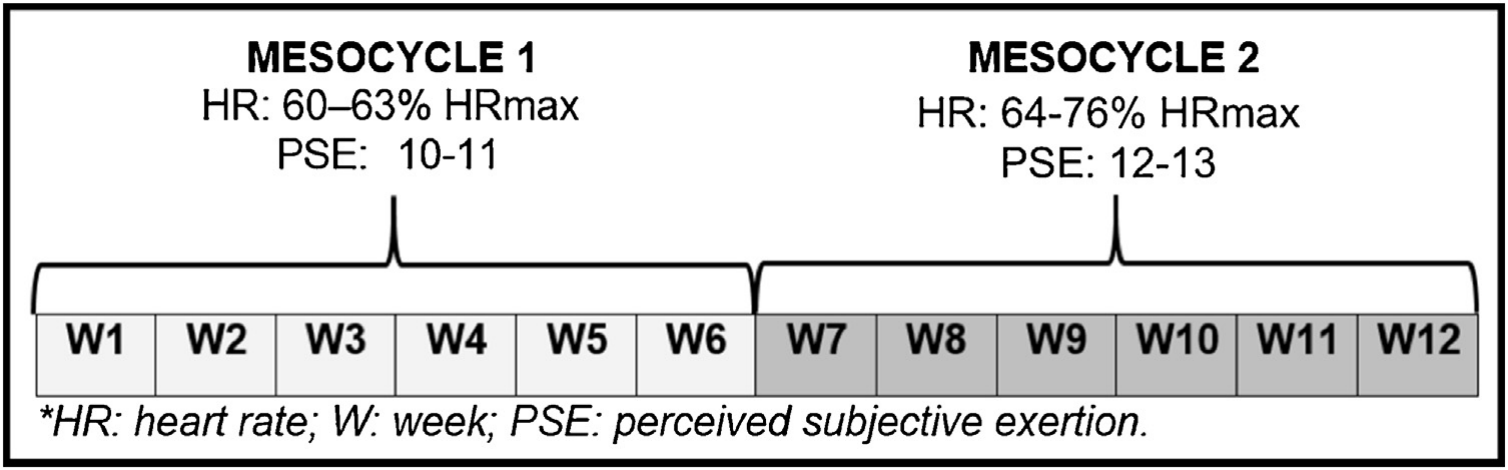
Training protocol.

Mesocycle 2 – Weeks 5 to 8: participants will cycle at an intensity eliciting HR between 64 and 76% of HRmax or 12 to 13 in the PSE scale, which ranges from 6 to 20. [16] (Fig. 3).

The exercise sessions will use the following routine: 1) place the heart monitor on the chest of the subjects; 2) place the BFR equipment on their thighs while standing; 3) inflate the pressurized band to induce BFR while sitting in HD chair; 4) place the stationary bicycle in front of the patient; and 5) instruct patients to cycle.

The maximum HR will be determined using the following formula: HRmax = 211-(0.64 x age) [17]. Patients using medications influencing HR, such as beta-blockers, will have their HR corrected based on the study of Godoy [18].

A cycle ergometer will be placed in front of the patient's chair and will be positioned at a distance allowing a relative knee angle (inner angle formed between the thigh and the leg) between 150° and 155° [19].

The BFRG will perform the exercise with BFR, while the CG will perform with no BRF. For the BFRG, a 6 cm wide inflatable band will be placed at the root of the lower limbs and inflated according to the thigh circumference of the patients, as suggested by Loenneke et al. [15]. The following thigh circumference/pressure will be applied to impose 50% of arterial restriction: from 45 to 50.9 cm: 100 mmHg; to 51 from 55.9 cm: 130 mmHg; from 56 to 59.9 cm: 150 mmHg; ≥60 cm: 180 mmHg. The patient will maintain BFR throughout the exercise. The exercise will be interrupted if the patient shows any discomfort or wants to stop.

### Study variables

The dependent variables of the study will be the health indicators (AAI, strength, functional capacity and quality of life), inflammatory variables (IL-6, IL-10 and CRP), oxidative stress (catalase, superoxide dismutase and glutathione peroxidase) and femoral quadriceps muscle thickness. The independent variable will be BFR, and the variables alcohol intake, diet, age, BMI and use of continuous medication, will be collected and considered in the analysis as possible confounders (Table 1).

**Table 1:** Dependent and independent study variables.

Variables	Definition	Scala	Operationalization
*Dependent*			
Interleukin 6	**Numeric**	**pg/ml**	---
Interleukin 10	**Numeric**	**pg/ml**	---
C-reactive protein	**Numeric**	**pg/ml**	---
Femoral quadriceps muscle thickness	**Numeric**	**mm**	---
Catalase activity	**Numeric**	**U/mg**	---
Superoxide dismutase activity	**Numeric**	**U/mg**	---
Glutathione peroxidase activity	**Numeric**	**U/mg**	---
Ankle-arm index	**Numeric**	**Ratio**	---
Functional test	**Numeric**	**Meters**	---
Strength	**Numeric**	**Lbs**	---
Quality of life	**Numeric**	**Score**	---
*Independent*			
Continuous moderate exercise intervention associated with partial blood flow restriction	Trichotomous	0, 1 or 2	**0 – Without exercise**
			**1- Exercise not using BFR**
			**2– Exercise using BFR**

BFR – Blood flow restriction.

#### Questionnaire

The questionnaire will consist of questions related to the following variables: age, sex, socioeconomic status, schooling, nutritional status, alcohol intake, continuous use medication, quality of life and 24-hour food recall. Continuous medicines use will be collected using an open-ended question asking whether the subjects use continuous medication and, if so, what the name of the drug is and the dosage taken. Quality of life will be evaluated using the Brazilian portuguese version of the Quality of Life Assessment of patients with CKD (KDQOL-SF™1.3) – instrument developed to evaluate the quality of life of individuals with CKD on dialysis [20]. It contains 79 items, 43 of which are related to CKD and 36 are related to general health condition.

### Data processing and analysis

The data will be doubled typed in EPIDATA 3.1 software, which will perform analysis to verify inconsistencies and correction of possible errors. The 5% significance level will be used and the statistical analysis will be conducted in Stata 14.0 software. The two mixed factors analysis of variance (ANOVA) will be used in the comparison of the training protocols, using Bonferroni as a post-hoc test for parametric variables. Kruskal-Wallis test and Dunn's post-hoc test will be used to non-parametric variables. The responsible researcher for the statistical analysis will be blinded regarding to the study objectives.

### Quality control

To assure the quality control of the interviews, the main researcher will re-apply some key questions (previously defined) to 10% of the interviewees. The answers will be used to detect possible problems in the interviewer’s performance. If there is a problem, all interviews will be repeated. In addition, to ensure quality control, blood samples will be analyzed in triplicate, data will be typed in duplicate in EpiData3.1 software, and follow-up meetings will be held weekly with the staff members who will check physical training.

### Ethical aspects

The study protocol was submitted to the Research Ethics Committee of the Catholic University of Pelotas and approved under No. 2,036,385. The following ethical principles will be assured to the participants. In addition, the subjects of the sample must sign an informed consent term. The study protocol was registered in the Brazilian Registry of Clinical Trials RBR-8T2P2M.

## Discussion

Individuals with CKD present altered inflammation and oxidative stress parameters, show a higher concentration of inflammatory markers and oxidizing enzymes, as well as a lower concentration of anti-inflammatory markers and antioxidant enzymes than healthy subjects [21], especially when exposed to therapy of renal replacement [22]. Studies aimed to verify the effects of exercise on patients submitted to HD are not new in the scientific literature. However, data from studies with physical exercise intervention on the immune system and oxidative stress in this population are rare and inconclusive. In addition, there is no consensus of exercise recommendations for patients submitted to HD [1].

The investigation of intradialytic exercise effects per se on clinical, physiological, functional and psychological parameters plays a significant role on the relevance of the present study. Furthermore, to our knowledge, this is the first RCT to investigate the impact of intradialytic exercise with BFR on the immune system and oxidative stress of individuals with CKD during HD. In addition, this RCT also aims to verify the effects of continuous moderate exercise either using or not BFR on the quality of life, functional capacity, muscle strength and muscle thickness in patients during HD, which are affected by CKD.

The main strengths of this study will be its randomized design and the inclusion of two different exercise protocols (cycling either using or not BFR) administered in a personalized way in patients in "real situation" who suffer from various adverse effects of CKD. As this RCT encounters the expected effects of continuous moderate exercise with BFR on the different studied variables, it would, at least, generate strong evidence for the implementation of an exercise program with BFR during HD to improve general health of this population.

Thus, it is expected that the present study will generate important information about the role of intradialytic continuous moderate exercise on the clinical, physical and psychological health of patients with CKD during HD. In addition, we aim to verify if performing exercise with BFR could optimize exercise results.

## Conflict of interests

Authors declare that there is no conflict of interest.
